# Functional characterization of two newly identified Human Endogenous Retrovirus coding envelope genes

**DOI:** 10.1186/1742-4690-2-19

**Published:** 2005-03-14

**Authors:** Sandra Blaise, Nathalie de Parseval, Thierry Heidmann

**Affiliations:** 1 Unité des Rétrovirus Endogènes et Eléments Rétroïdes des Eucaryotes Supérieurs, UMR 8122 CNRS, Institut Gustave Roussy, 39 rue Camille Desmoulins, 94805 Villejuif Cedex, France; 2 Unité de Biologie des Rétrovirus, Département de Virologie, Institut Pasteur, 25 rue du Dr Roux, 75724 Paris cedex 15, France

## Abstract

A recent *in silico *search for coding sequences of retroviral origin present in the human genome has unraveled two new envelope genes that add to the 16 genes previously identified. A systematic search among the latter for a fusogenic activity had led to the identification of two *bona fide *genes, named syncytin-1 and syncytin-2, most probably co-opted by primate genomes for a placental function related to the formation of the syncytiotrophoblast by cell-cell fusion. Here, we show that one of the newly identified envelope gene, named *env*P(b), is fusogenic in an *ex vivo *assay, but that its expression – as quantified by real-time RT-PCR on a large panel of human tissues – is ubiquitous, albeit with a rather low value in most tissues. Conversely, the second envelope gene, named *env*V, discloses a placenta-specific expression, but is not fusogenic in any of the cells tested. Altogether, these results suggest that at least one of these *env *genes may play a role in placentation, but most probably through a process different from that of the two previously identified syncytins.

## Findings

**Figure 1:**
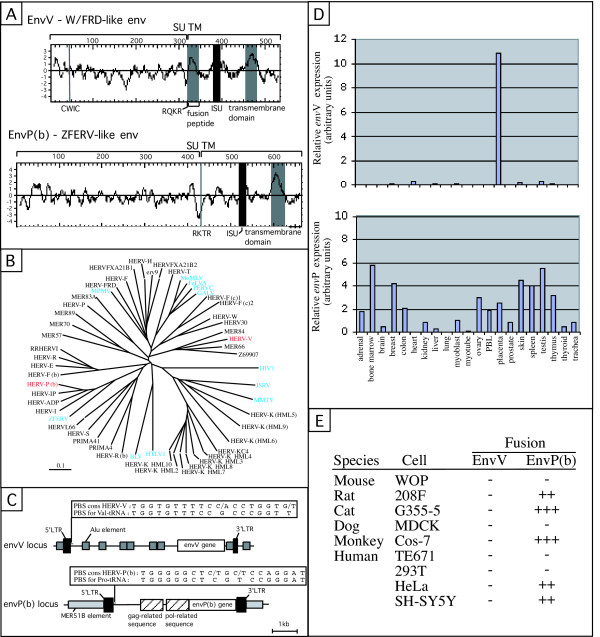
A) Hydrophobicity profile and predicted features of the EnvV (formerly W/FRD-like env) and EnvP(b) (formerly ZFERV-like env) proteins. The SU (Surface Unit) and TM (TransMembrane) moieties of the envelopes are delineated, with the position of the putative proteolytic cleavage site (consensus, R/K-X-R/K-R) between the two subunits and the « CWLC » motif (consensus, C-X-X-C) indicated. The hydrophobic regions associated with the fusion peptide and the transmembrane region are shaded in light gray, and the putative immunosuppressive domain (ISU) in dark gray. B) Phylogenetic tree of retroviral envelopes and position of the newly identified genes. The tree is based on an alignment of approximately 180 amino acids corresponding to the extracellular and transmembrane domains of the TM subunit of envelope proteins. The protein alignment, phylogenetic tree and bootstrap analysis were performed with the ClustalW program (neighbour joining option). The tree was viewed by using the TreeView program. The scale bar indicates 10% aa sequence difference. The phylogenetic tree determined by the parsimony method was congruent with the neighbour joining tree (data not shown). The two "new" V and P(b) *env *genes are represented in red, ERV *env *genes from other species and exogenous retroviruses in blue. The sequences used for the alignments were those of the consensus element of each family, or the coding *env *gene when present. The consensus sequences of the HERVK(HML-9), HERVFXA21B1 and HERVFXA21B2 families, which are not listed in Repbase, were each inferred from the comparison of 3–6 sequences. Abbreviations: MoMLV, Moloney Murine Leukemia Virus; FeLVA, Feline Leukemia Virus strain A; PERVC, Pig Endogenous Retrovirus strain C; GALV, Gibbon Ape Leukemia Virus; MPMV, Mazon-Pfizer Monkey Virus; MMTV, Mouse Mammary Tumor Virus; JSRV, Jaaksiekte Sheep Retrovirus; HTLV, Human T cell leukemia Virus; BLV, Bovine Leukemia Virus; HIV, Human Immunodeficiency Virus. C) Genomic organization of the *env*V and *env*P(b) loci. The envelope ORF (open box) with *gag- *and *pol- *related sequences (hatched boxes) and long terminal repeats (black boxes), Alu (dark gray boxes) and MER51B (light gray boxes) retroelements are indicated. Consensus PBS sequences (obtained from two sequences for the HERV-V family and from four sequences for the HERV-P(b) family) are indicated above the corresponding provirus, together with the PBS for the Val and Pro-tRNA, respectively. D) *env*V and *env*P(b) mRNA expression in a panel of 19 healthy human tissues, as determined by real-time quantitative RT-PCR. RNAs from human tissues were prepared as described in [6]. The reaction was performed using Sybr Green Master Mix (Applied Biosystems). PCR was developed using an ABI PRISM 7000 sequence detection system. Primer sequences (5'-3') were as follows: (CATGACTTTGGAAAAGGAGG) and (GCCAAAGAGGAAAAGTAAGAGT) for *env*V; (CAAGATTGGGTCCCCTCAC) and (CCTATGGGGTCTTTCCCTC) for *env*P(b). The transcript levels were normalized relative to the amount of 18S mRNA (as determined with the primers and TaqMan probe from Applied Biosystems). Samples were assayed in duplicate. PBL, peripheral blood lymphocytes. E) Assay for fusogenicity of *env*V and *env*P(b). XhoI containing primer sequences (5'-3') were as follows: (ATCACCTCGAGACACTCCATCGAACCACTTCAT) and (ATCACCTCGAGGGCTGTTCTAGGATGGGTTATT) for *env*V; (ATCACCTCGAGAGAAGAGAAACTTGAACCGTCC) and (ATCACCTCGAGGGGCTGATAGATGAATGGGTAT) for *env*P(b). The PCR products were cloned into the phCMV-G vector, opened with XhoI, and the constructs were verified by partial sequencing. Cell lines and fusion assays are as described in [12], except for the SH-SY5Y neuroblastoma cell line (ATCC number CRL-2266).

Endogenous retroviral sequences represent approximately 8% of the human genome. These sequences (called HERVs for Human Endogenous Retroviruses) share strong similarities with present-day retroviruses, and are the proviral remnants of ancestral germ-line infections by active retroviruses, which have thereafter been transmitted in a Mendelian manner (reviewed in [1-3]). The 30,000 HERV elements have been grouped according to sequence homologies into more than 80 distinct families (each originating from the same founder element), based on a systematic listing of human repeats in the Repbase database [4]. Most of these elements are non-coding due to the accumulation of mutations, deletions, and/or truncations. A screening of the human genome for retroviral envelope genes with coding capacity, based on a specific envelope protein motif and on the HERV families described in Repbase, has revealed 16 fully coding envelope genes, transcribed in several healthy tissues [5,6], among which two (syncytin-1 and syncytin-2) possess a fusogenic activity [7,8]. Using another approach, based on BLAST searches with various retroviral sequences as queries, a recent elegant study has analyzed the coding potential of human retroviral sequences and two additional fully coding envelope genes have emerged from this screen [9]. These two envelope genes do not belong to the HERV families listed in Repbase. The first one was designated "HERV-W/FRD-like" *env*, due to partial homology with syncytin-1 and syncytin-2, encoded by proviruses of the HERV-W and HERV-FRD families, respectively [7,8]. The second one was designated "ZFERV-like" *env*, due to its homology with the envelope protein encoded by a provirus recently discovered in the zebrafish genome [10]. The sequences and predicted hydrophobic profiles of the two proteins (renamed here EnvV and EnvP(b) respectively, see below), disclose the characteristic signature of retroviral envelope proteins, with a putative proteolytic cleavage site between the SUrface (SU) and TransMembrane (TM) moieties, and a hydrophobic transmembrane domain within the TM subunit which permits its anchorage to the membrane (Figure 1A).

Since these genes belong to previously uncharacterized HERV families, we first analyzed their phylogenetic relationship with known HERV families and animal retroviruses. We generated a phylogenetic tree of endogenous and exogenous retroviruses based on the *env *gene, namely on the alignment of a conserved domain of the transmembrane (TM) subunit [3,5]. In this tree (Figure 1B), the "HERV-W/FRD-like" *env *gene is closely related to that of MER66, MER84 and Z69907 families. This gene seems to be part of a very degenerate proviral structure, with only the LTR being identifiable (see below and Figure 1C). As mentioned in [9], a highly homologous gene (95.7% identity at the nucleotide level) encoding an envelope protein truncated due to a frameshift can be found 40 kb downstream. This cognate *env *gene is unambiguously part of a proviral structure, displaying just upstream of it the 1.6 kb open reading frame of a *gag *gene, followed by a *pol*-like non coding region (data not shown. The flanking sequences of both proviruses are distinct. No other provirus or *env *gene belonging to this "family" can be found in the human genome by a BLAST search on the Ensembl database. Approximately 4 kb upstream of each of these two *env *genes, as expected, the RepeatMasker program that screens DNA sequences for interspersed repeats present in mammalian genomes  identifies 5' LTR sequences (or fragments of LTR sequences). 3' LTRs are also found just downstream of the envelope genes (see Figure 1B for the map of the fully coding *env *gene locus). The analysis of the PBS (Primer Binding Site) region located downstream of the two 5' LTRs of this family reveals a high degree of homology to the PBS for Val-tRNA (Figure 1C), so we propose to name this new family HERV-V.

The "ZFERV-like" *env *gene clusters, in the TM-based tree, with the "HERV-I superfamily", which indeed also includes the ZFERV *env *from zebrafish (see Figure 1B). As indicated in the retrosearch database , this envelope gene is part of an identifiable provirus (see Figure 1C). A BLAST query on the Ensembl database using the provirus sequence showed that this new HERV family contains three additional members. All four HERV elements, harbouring a proviral LTR-gag-pol-env-LTR structure (although the only coding gene is the *env *gene described in [9]), are close to – but yet unambiguously distinct from – the HERV-IP family. The analysis of the PBS region of these four proviruses reveals a high degree of homology to the PBS for Pro-tRNA (see Figure 1C), so we propose to name this new family HERV-P(b) (since the HERV-P family already exists, [11]).

To determine whether these two genes could play a role in human placentation, we then characterized their expression pattern and fusogenic properties, as previously performed for the 16 coding envelope genes already identified [6,8]. To get insight into their expression profile, we used a Real-Time RT-PCR strategy as described in [6]. In this study, specific primers had been designed for Sybr Green amplification in such a way that only *env *genes with an open reading frame would be amplified among all the envelope genes of a given family, by positioning them within domains of maximal divergence between the coding and the non-coding copies. For the HERV-V coding envelope, the primer pair was designed in the 3' part of the gene, where the two *env*V genes are the most divergent (79% identity in the last 200 nt). An additional primer pair was also designed to monitore the expression of the truncated HERV-V *env *gene. To assess the specificity of each primer pair for the corresponding *env *gene, the PCR products obtained upon amplification of genomic DNA were cloned into a pGEM-T vector and 6 clones per amplicon were sequenced. In each case, the 6 sequences corresponded to the expected *env *gene. Analysis of the expression level of the coding *env*P(b) and *env*V genes was achieved on a series of 19 healthy human tissues, and the results are represented in Figure 1D. The expression pattern of *env*V was found to be placenta-specific. Interestingly, the truncated envelope of the HERV-V family is highly expressed in the placenta as well, but poorly in other tissues (data not shown). *Env*P(b) expression, on the other hand, was observed at a rather low level in almost all the tissues tested, without any specificity for the placenta.

Among the 16 coding *env *genes of the human genome tested in [8], only two, namely *env*W (syncytin-1) and *env*FRD (syncytin-2), had been found to be fusogenic in an *ex vivo *assay. As these two *env *genes were highly and specifically expressed in the placenta, it was suggested that they are involved in a major physiological process within this organ, namely fusion of the cytotrophoblast cells to form the syncytiotrophoblast layer. The two newly identified *env *genes were therefore similarly tested. To do so, they were first cloned and introduced into a eukaryotic expression vector. The *env*P(b) gene was PCR-amplified from the DNA of BAC RP11-828K24 by using a proofreading DNA polymerase and running a 15-cycle PCR reaction, whereas the *env*V gene -not available as BAC DNA- was PCR amplified from the genomic DNA of a Caucasian individual using the Expand long template enzyme mix (Roche Applied Science). Both *env *genes were then assayed for cell-cell fusion on a large panel of mammalian cells (known to express on the whole the receptors for all retroviral envelopes identified to date) using a transient transfection assay and two clones from each construct. As shown in Figure 1E, cell-cell fusion was observed in five out of nine cell lines tested for *env*P(b), and in none of them for *env*V. The truncated envelope protein member of the HERV-V family was also tested and, as expected, was not fusogenic (data not shown). In some respect, these results are surprising. Indeed, the putative protein encoded by *env*P(b) is fusogenic despite the absence of a canonical fusion peptide, i.e. of a hydrophobic region located at the N-terminus of the putative TM subunit, just downstream of the SU-TM cleavage site (see Figure 1A). Conversely, the *env*V gene product, notwithstanding its canonical sequence, is not fusogenic (at least in the panel of cells tested). To check that the lack of fusogenicity of the latter gene is not due to a fortuitous gene polymorphism of the *env*V gene from the selected individual, we PCR-amplified, cloned and assayed the *env*V gene from two other individuals (for both the complete and the truncated *env*V genes): no cell-cell fusion was observed either (data not shown). Finally, we identified and cloned the chimpanzee orthologous *env*V gene (which is fully coding as well): neither did it display any fusogenic activity in our assay (data not shown).

In conclusion, the present analysis shows, rather paradoxically, that the envelope protein with fusogenic properties is not placenta-specific, whereas the one which is exclusively expressed in the placenta -a characteristic pattern of the two previously described fusogenic syncytin-1 and syncytin-2 gene products- is not fusogenic. In this respect, these results suggest that the two newly identified *env*V and *env*P(b) genes are most probably not "syncytin-like" genes, *sensu stricto*. Additional experiments should now be devised (e.g. search for conservation among primates, search for Single Nucleotide Polymorphisms) to assess their role -if any- in human physiology.

## List of abbreviations

HERV, human endogenous retrovirus; TM, transmembrane; LTR, Long Terminal Repeat; PBS, Primer Binding Site.

## Competing interests

The author(s) declare that they have no competing interests.

## Authors' contributions

SB carried out the cloning of the *env *genes and the cell-cell fusion assays.

NdP analyzed the sequences, constructed the phylogenetic tree, designed and carried out the Real-Time RT-PCR experiments, and drafted the manuscript.

TH conceived the study.
